# Left Ventricular Noncompaction as a Rare Cause of Syncope

**DOI:** 10.7759/cureus.19409

**Published:** 2021-11-09

**Authors:** Pius E Ojemolon, Endurance O Evbayekha, Jesse Odion, Jeremiah Bello, Hafeez Shaka

**Affiliations:** 1 Internal Medicine, John H. Stroger, Jr. Hospital of Cook County, Chicago, USA; 2 Internal Medicine, Stella Obasanjo Isolation Centre, Benin, NGA; 3 Internal Medicine, University of Benin Teaching Hospital, Benin, NGA; 4 Medicine, University of Benin, Benin City, NGA

**Keywords:** arrhythmia, heart failure, thromboembolism, congenital, cardiomyopathy, noncompaction

## Abstract

Left ventricular noncompaction (LVNC) is a relatively rare myocardial disorder which is characterized by trabeculations and deep intertrabecular recesses within the left ventricle. LVNC is often asymptomatic but may present with heart failure, arrhythmias, or systemic thromboembolism. Uncommonly, patients with LVNC can present with syncope. In this article, we report one such presentation of this rare medical condition.

## Introduction

Left ventricular noncompaction (LVNC) is an uncommon myocardial disorder characterized by trabeculations and deep intertrabecular recesses within the left ventricle. There is a strong genetic component to the disorder and 30%-50% of cases are familial [1-3].

The clinical presentation of patients with LVNC varies from asymptomatic to heart failure, arrhythmias, systemic thromboembolism, and even sudden cardiac death (SCD). Cardiac syncope is a rare presentation. With the advent of new high-resolution diagnostic imaging techniques, more cases of LVNC are being detected. However, misdiagnosis/late diagnosis is still quite common, as the clinical manifestations of the disease are variable and may be explained by more common conditions [1,2].

We present an uncommon presentation of this rare medical condition along with a brief review of available literature on it.

## Case presentation

An African American gentleman in his early 40s with a history of hypertension, heart failure with reduced ejection fraction, and tobacco use disorder, as well as a family history significant for unspecified heart disease in his mother, presented to the emergency department with recurrent chest pain, shortness of breath, and two syncopal episodes spaced two days apart. The chest pain was sharp, retrosternal, mild, non-radiating, non-exertional, with frequent recurrence, and associated with palpitations. His shortness of breath was exertional but improved following a recent admission for decompensated heart failure. Following his recent discharge, he noted an episode of dizziness associated with palpitations and transient loss of consciousness. He had a similar episode while on a train ride two days later. He could not determine the duration for which he was passed out but denied confusion, tongue biting, and bowel or bladder incontinence.

He was diagnosed with heart failure with reduced ejection fraction (left ventricular ejection fraction of 20%-25%) eight months prior and was placed on guideline-directed medical therapy with carvedilol and losartan. The patient had multiple prior emergency visits for chest pain, which had been deemed non-anginal with recent left heart catheterization demonstrating no evidence of coronary artery disease. He however had poor outpatient follow up and poor medication adherence. 

**Figure 1:**
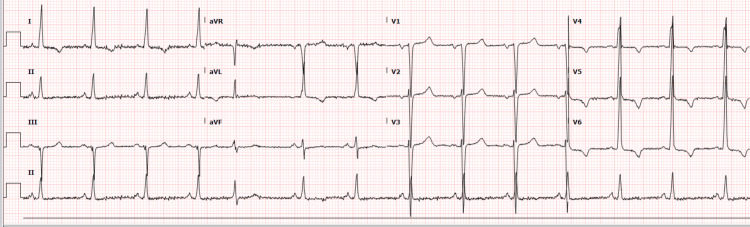
ECG showing normal sinus rhythm with occasional ectopic premature complexes, normal axis, left and right atrial enlargement, left ventricular hypertrophy, and T wave inversions in leads aVL and V4-6

Vital signs on presentation included a mildly elevated blood pressure of 138/108 mmHg, heart rate of 86 beats per minute, respiratory rate of 18 cycles per minute, temperature of 36.4 degrees Celsius, and oxygen saturation of 100% in room air. Physical examination was significant for absent jugular venous distention, clear lungs to auscultation, and trace pedal edema. Electrocardiography (ECG) on presentation showed normal sinus rhythm with occasional ectopic premature complexes, normal axis, left and right atrial enlargement, left ventricular hypertrophy, and T wave inversions in leads aVL and V4-6 representing left ventricular strain pattern, unchanged compared to prior ECGs (Figure 1). Laboratory investigations including serum electrolytes, blood urea nitrogen, serum creatinine, complete blood count, and cardiac enzyme panel were without gross derangements. Specifically, troponin-I on presentation was 0.02 ng/mL (normal range <0.028 ng/mL) and B-type natriuretic peptide (BNP) was 73 pg/mL (normal range <100 pg/mL).

**Figure 2:**
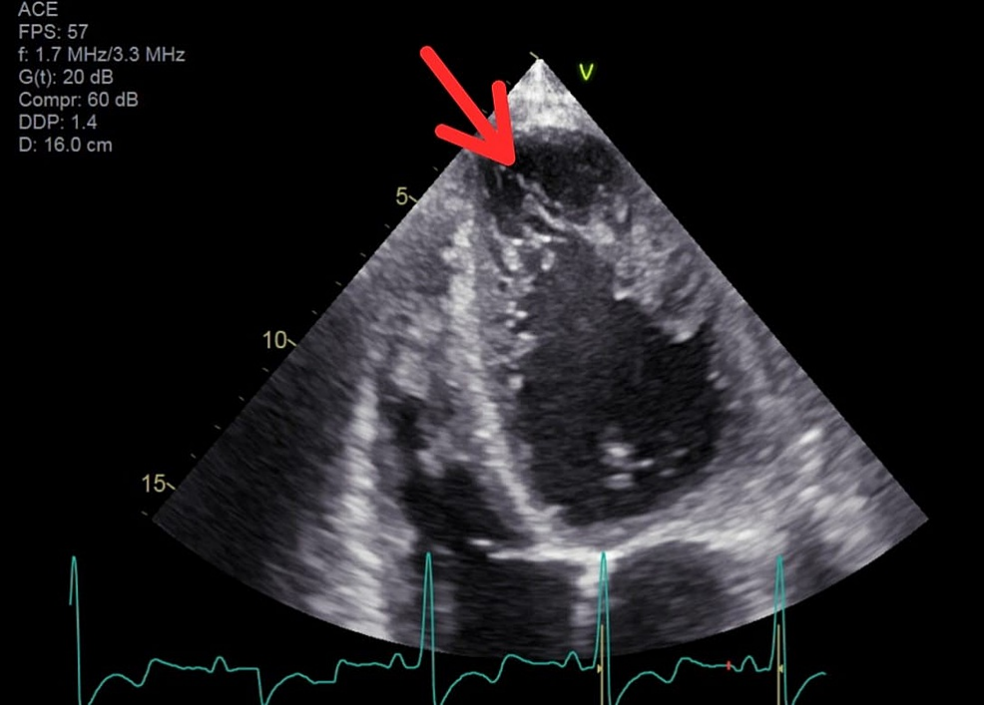
Apical four-chamber view on transthoracic echocardiography showing prominent left ventricular wall trabeculations The red arrow highlights the area of non-compacted left ventricular myocardium with trabeculations.

**Figure 3:**
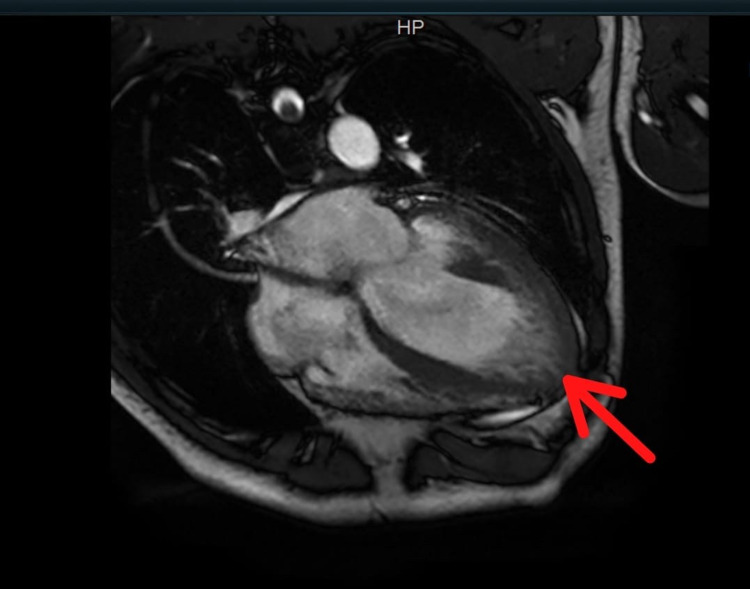
Cardiac MRI revealed a severely dilated left ventricle with increased trabeculations within the mid to apical inferior, mid to apical lateral and true apex (highlighted by the red arrow)

**Figure 4:**
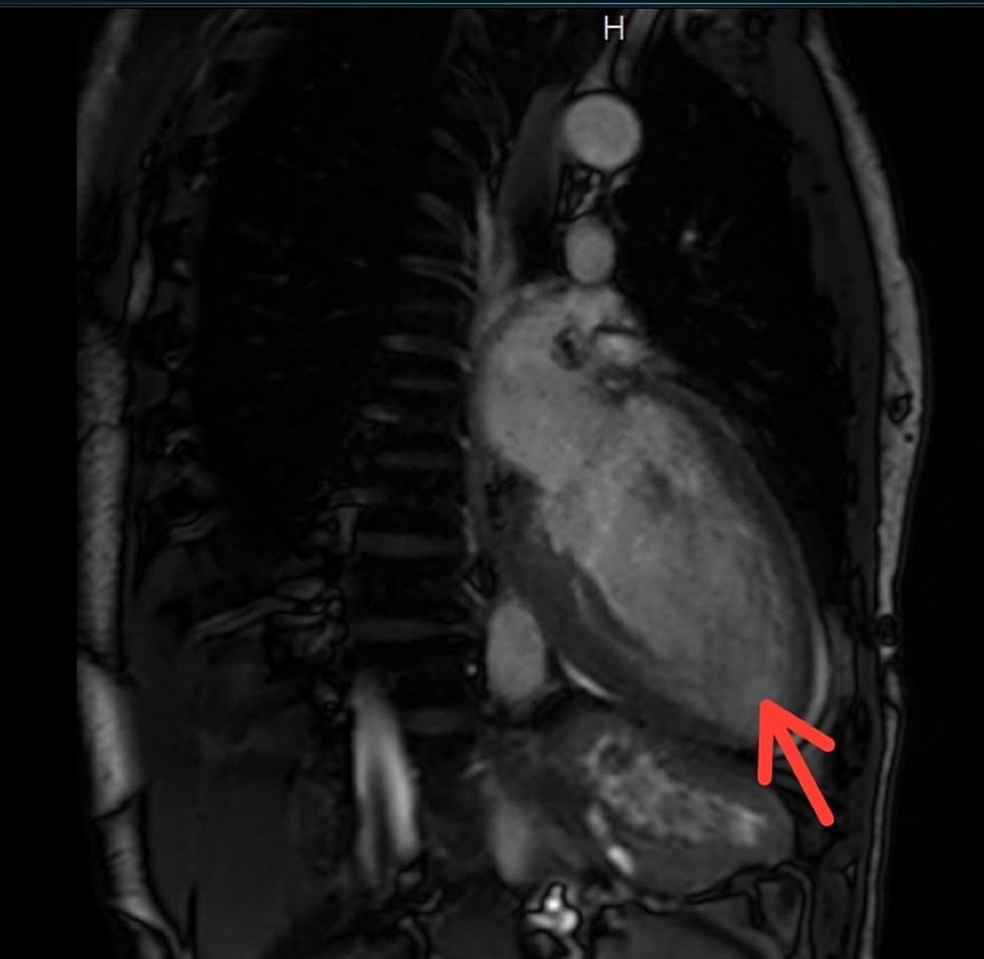
Cardiac MRI revealed a severely dilated left ventricle with marked noncompaction and prominent trabeculations in the region of the true apex The red arrow highlights the area of non-compacted apical left ventricular myocardium.

He was admitted for further workup and placed on telemetric monitoring. Overnight telemetry revealed multiple runs of non-sustained ventricular tachycardia. Transthoracic echocardiography showed an estimated ejection fraction of 20%-25% (similar to his baseline from eight months prior), severe diffuse hypokinesis with regional variations as well as prominent trabeculations of the left ventricular wall suggestive of LVNC (Figure 2). Cardiac magnetic resonance imaging (MRI) was ordered which revealed a severely dilated left ventricle with increased trabeculations within the mid to apical inferior, mid to apical lateral, and true apex. There was myocardial thinning along the left ventricular apex. Maximal ratio of trabeculated non-compacted myocardium to compacted myocardium wall measured approximately 2.6 in the lateral/inferolateral wall in end-diastole. Right ventricular size, shape, function, and diastolic wall thickness were within normal limits. On the delayed post-contrast sequences, there was a thin stripe of mid-myocardial enhancement in the basal to mid anterior wall and basal to mid septum. In addition, there was focal hyperenhancement of the posteromedial papillary muscle (Figures 3-4).

He was counseled extensively on the importance of compliance with his medications and was recommenced on guideline-directed medical therapy (same regimen as above) before being discharged to follow up with a cardiac electrophysiologist. He was also counseled for and subsequently had an automatic implantable cardioverter-defibrillator (ICD) placed. A recent device interrogation revealed an episode of sustained ventricular tachycardia lasting over 40 seconds which was not associated with symptoms.

## Discussion

LVNC was first described in 1926 but was only classified as a distinct cardiomyopathy by the American Heart Association in 2006. The prevalence of LVNC has been estimated to vary from 0.01% to 1.3% in patients referred for echocardiography. However, recent studies show that it may be seen in as high as 3%-4% of patients with heart failure [3-5].

LVNC often affects the apex, mid-ventricular inferior wall, and the mid-ventricular lateral wall of the left ventricle. In some patients, the right ventricular apex may be affected as well. The myocardium in LVNC has a “spongy” appearance characterized by a thin, compacted epicardial layer and a non-compacted endocardial layer, with prominent trabeculae and deep intertrabecular recesses that communicate with the left ventricular cavity. This is thought to result from premature cessation of the compaction of the loosely interwoven meshwork of myocardial fibers during endomyocardial morphogenesis, a process that occurs between 12-18 weeks of fetal life, beginning at the base of the left ventricle and progressing toward the apex. This process of trabeculation appears to involve the release of neuregulin growth factors from the endocardium and may also involve angiogenetic factors, such as vascular endothelial growth factor and angiopoietin-1 [4-8].

LVNC may be sporadic or familial with an autosomal dominant or X-linked mode of inheritance. It may be seen in isolation but is often associated with other congenital heart defects, mitochondrial inheritance diseases, or musculoskeletal disorders. Although only a minority of patients have been successfully genotyped, several genes have been implicated in the development of LVNC including α-dystrobrevin (DTNA), LIM domain-binding protein 3 (ZASP), tafazzin (TAZ/G4.5), and those encoding sarcomeric, Z-disc, cytoskeletal, and mitochondrial proteins. Echocardiographic screening is recommended for first-degree relatives of patients with LVNC [7-11].

Age of onset and degree of severity depend on the extent of the non-compacted cardiac segments. The clinical presentation of LVNC ranges from absence of symptoms to congestive heart failure, arrhythmias (typically atrial fibrillation and ventricular tachyarrhythmias), thromboembolic events, and SCD. Heart failure is the most common clinical manifestation of LVNC and is the initial presentation in more than half of affected patients. In advanced cases, the classic triad of heart failure, ventricular arrhythmias, and thromboembolic events may be present. Uncommonly, patients can present with recurrent syncope. Syncope in LVNC patients is typically associated with palpitations (as seen in our case), suggesting that the syncope is cardiogenic, arising due to arrhythmias. This is buttressed by in-hospital telemetry for our patient showing evidence of multiple runs of non-sustained ventricular tachycardia and automated implantable cardiac defibrillator device interrogation post-discharge revealing an episode of sustained ventricular tachycardia. The prevalence of arrhythmias in LVNC patients remains unclear. However, they are thought to be significantly more common than in the general population, particularly in those who have already developed heart failure (a potent trigger for malignant arrhythmias). Furthermore, recurrent syncope in LVNC may be predictive for eventual SCD [1,4-7,12].

Early diagnosis is of paramount importance due to associated high morbidity and mortality. ECG findings include left ventricular hypertrophy, conduction abnormalities, ventricular arrhythmias, and T-wave inversions. The condition is typically diagnosed by echocardiography and/or cardiac MRI showing large prominent trabeculae and deep intertrabecular recesses in which flow can be detected. Furthermore, the bi-layered myocardial wall structure (thin, compacted epicardium and thick, non-compacted endocardium) may be visualized. Cardiac MRI holds particularly significant value in the diagnosis of LVNC because of its ability to visualize the endocardium. A noncompaction/compaction ratio of >2.3 in diastole has been used to distinguish pathological noncompaction. MRI also has the added ability to clearly visualize the apex, which has been a limitation of echocardiography. LVNC has also been identified by high-resolution computed tomography and positron emission tomography [2,4,6-8,13].

Outcomes and appropriate therapies remain poorly defined, thus the management of LVNC is dynamic and targeted at the relevant symptomatology. Heart failure is managed with medical therapy, but end-stage heart failure may require heart transplantation. Systemic anticoagulation is often used to reduce the formation of left ventricular thrombi and thromboembolic events. For the high risk of atrial and ventricular arrhythmias, patients are often monitored with periodic ambulatory ECG monitoring to detect asymptomatic arrhythmias, especially in patients with systolic dysfunction. Expanded monitoring by a loop recorder may provide better information. Electrophysiological study (EPS) can be used to determine the risk for ventricular arrhythmias and SCD. ICD may be considered for the management of ventricular arrhythmias. ICDs are often indicated in cases of aborted cardiac arrest, sustained ventricular tachycardia, exertional syncope related to ventricular arrhythmias, family history of SCD, or severely impaired left ventricular ejection fraction [2,5-8].

Asymptomatic patients generally carry a favorable prognosis. Predictors of increased mortality include increased age, neuromuscular disorders, severe arrhythmias, New York Heart Association (NYHA) class III/IV heart failure, exertional syncope related to ventricular arrhythmias, permanent atrial fibrillation, bundle-branch block, left ventricular dilatation/dysfunction, and thromboembolic events [5-9,14,15].

## Conclusions

While improvements in cardiac imaging have led to recent increases in the prevalence of LVNC, the correct diagnosis is still often missed/delayed because of lack of knowledge about this uncommon disease and its similarity to other diseases of the myocardium and endocardium. LVNC should be considered in the differential diagnosis of recurrent syncope, particularly in patients with heart failure and tachyarrhythmias.
